# The Motherless

**Published:** 1878-07

**Authors:** 


**The Motherless.**—They are *motherless!* Oh! gently, gently keep back those bitter words. Avert that cold, cruel stare. See you not the tearful eyes? Alas! that sorrow should ever make a child's heart its home!

They are motherless! Stranger hands ministering to their daily wants; stranger hearts wearying of the irksome duty.

No fond, sweet kisses of warm embrace! No gentle words of comfort and love! No soft folding of little hands in prayer! No *mother!*

Missing the low, sweet cadence of her voice; missing that "Good-night!" seeking, seeking all in vain, that ark for the weary dove—a mother's *heart.*

Draw the little forms near to your heart. Pillow the aching head upon your bosom. Think of your sunny childhood—your mother's earnest love, her gentle care, her patient forbearance, her precious forgiveness. Then only in kindness let your hand rest on each honored little head; only in love reprove that stricken little flock.

Oh! let yours be the hand that will lead them in the green pastures, and by the still waters of the precious Saviour's love! Let yours be the blessed benediction: "Inasmuch as ye have done it to the least of these, ye have done it unto me." Remember their angels do always behold the face of our Father in heaven. Then, it may be that a child's hand shall lead you to that heavenly home—a child's hand place the crown upon your head.

Speak gently to the motherless!
  A weight of woe they bear;
Greet them with looks of tenderness—
  Oh! add not to their care.
Speak gently to the motherless
  When tears their eyes bedim;
Remember who has bid them "come,"
  And lead them unto Him.
Then yours shall that blessing be—
"Friends ye have done this unto me!"

